# Cognitive, Genetic, Brain Volume, and Diffusion Tensor Imaging Markers as Early Indicators of Dementia

**DOI:** 10.3233/JAD-200445

**Published:** 2020-10-13

**Authors:** Theresa Müller, Nicola M. Payton, Grégoria Kalpouzos, Frank Jessen, Giulia Grande, Lars Bäckman, Erika J. Laukka

**Affiliations:** a Aging Research Center, Department of Neurobiology, Care Sciences and Society, Karolinska Institutet and Stockholm University, Stockholm, Sweden; b University of Cologne, Faculty of Medicine and University Hospital Cologne, Germany; c German Center for Neurodegenerative Diseases (DZNE), Bonn, Germany; d Stockholm Gerontology Research Center, Stockholm, Sweden

**Keywords:** *APOE*, cognition, diffusion tensor imaging, magnetic resonance imaging, preclinical dementia, white matter

## Abstract

**Background:**

Although associated with dementia and cognitive impairment, microstructural white matter integrity is a rarely used marker of preclinical dementia.

**Objective:**

We aimed to evaluate the individual and combined effects of multiple markers, with special focus on microstructural white matter integrity, in detecting individuals with increased dementia risk.

**Methods:**

A dementia-free subsample (*n* = 212, mean age = 71.33 years) included in the population-based Swedish National Study on Aging and Care (SNAC-K) underwent magnetic resonance imaging (T1-weighted, fluid-attenuated inversion recovery, diffusion tensor imaging), neuropsychological testing (perceptual speed, episodic memory, semantic memory, letter and category fluency), and genotyping (*APOE*). Incident dementia was assessed during six years of follow-up.

**Results:**

A global model (global cognition, *APOE*, total brain tissue volume: AUC = 0.920) rendered the highest predictive value for future dementia. Of the models based on specific markers, white matter integrity of the forceps major tract was included in the most predictive model, in combination with perceptual speed and hippocampal volume (AUC = 0.911).

**Conclusion:**

Assessment of microstructural white matter integrity may improve the early detection of dementia, although the added benefit in this study was relatively small.

## INTRODUCTION

A major focus in dementia and Alzheimer’s disease (AD) research involves detecting people in a prodromal phase. This is essential as early interventions may delay clinical onset, slow down disease progression [1], and decrease the number of individuals affected by the disease [2]. In contrast to most previous research, this study concerns older people recruited from the general population and, thus, was not restricted to individuals with mild cognitive impairment (MCI) attending a memory clinic. We aimed to assess the added benefit of including markers from different modalities in dementia prediction, with special focus on markers of microstructural white matter integrity, which are rarely examined in this context.

Preclinical signs of dementia and AD can be detected many years before a clinical diagnosis may be rendered [3]. In the cognitive domain, global cognitive ability, perceptual speed, executive function, and episodic memory are frequently affected [4]. Although most studies in this area have targeted AD, similar patterns of cognitive deficits have been observed in preclinical vascular dementia (VaD) [5]. It should be noted, however, that in an older dementia sample, most cases can be expected to be a mixture between AD and VaD, whereas pure VaD is particularly rare [6].

Alongside cognitive deficits, there are numerous other risk factors and biological markers of dementia. Carrying at least one *APOE* *ɛ*4 allele is the major genetic risk factor for developing AD [7] and may therefore add important information, especially when combined with other dementia markers [8]. In the field of structural magnetic resonance imaging (MRI), hippocampal volume has been shown to be a particularly useful predictor of incident AD [9].

Besides being associated with brain atrophy, dementia and AD is accompanied by decline in white matter microstructure [10]. Reduced white matter integrity, as assessed with diffusion tensor imaging (DTI), has been shown to predict conversion to amnestic MCI among cognitively normal individuals [11] and has been associated with higher conversion rates from MCI to AD [12]. A recent largescale study revealed associations between overall mean diffusivity and incident MCI and dementia [13]. Furthermore, reduced white matter integrity has been associated with carrying the *APOE* *ɛ*4 allele [14] and having poorer perceptual speed performance in healthy adults [15, 16].

An advantage of assessing microstructural white matter integrity is that reductions are present early in dementia development relative to other structural brain changes, potentially before brain atrophy and white matter hyperintensities (WMH) can be detected [17–19]. Thus, DTI may add useful information in the early detection of future dementia and reveal initial structural brain changes. The earliest microstructural white matter alterations in AD start in the limbic tracts, after which they extend to lateral temporoparietal association fibers and long-ranging association tracts that also involve frontal white matter [20]. Deterioration of white matter tracts connecting structures important for memory, such as the cingulum and inferior fronto-occipital fasciculus, is thought to be particularly associated with higher risk of developing AD [21, 22].

A study by Mielke et al. [23] found DTI-derived measures of white matter integrity of the fornix to be highly predictive of progression to AD among participants with MCI (AUC > 90%), comparable to the predictivity of hippocampal volume. Another study, directly comparing the predictive abilities of brain volume and microstructural white matter integrity, found that mean diffusivity in the right basal forebrain was associated with increased conversion from MCI to AD. However, in a combined model, only hippocampal volume was significantly associated with conversion to dementia [24]. Thus, it is important to evaluate the relative usefulness and added value of indicators of microstructural white matter integrity in early detection of dementia in relation to other preclinical markers.

Including markers from more than one modality (e.g., neuropsychology, neuroimaging) may lead to improved prediction of future dementia and AD [25, 26]. We have previously evaluated the combined effect of cognitive, genetic, and neuroimaging markers in early detection of dementia [27]. In the current study, we focus on a subsample that also underwent a DTI sequence. We aimed to investigate the usefulness of both global and specific markers of dementia, alone and in combination, with special emphasis on markers of microstructural white matter integrity.

## METHODS

### Participants

The population-based Swedish National Study on Aging and Care in Kungsholmen (SNAC-K) started in 2001. Participants belonged to eleven age cohorts (60, 66, 72, 78, 81, 84, 87, 90, 93, 96 years, and 99 years and older) and were randomly selected based on their date of birth. In the present study, a subsample of the SNAC-K magnetic resonance imaging (MRI) sample (*n* = 555) that had also a DTI sequence (*n* = 260) was analyzed [28]. Due to exclusion (poor image quality/technical issues: *n* = 17, infarct/meningioma: *n* = 6, missing cognitive data: *n* = 7) and drop out (*n* = 18), the final analytical sample consisted of 212 participants. Of these, 173 remained dementia free, 16 developed dementia, and 23 died during the six-year follow-up. Compared to the original sample, including all SNAC-K participants (*n* = 3,363), this sample was significantly younger (71.33±8.76 years versus 73.71±10.69 years; *p* = 0.002) and performed significantly better on the Mini-Mental State Examination (MMSE) [29] at baseline (29.15±1.02 versus 27.01±5.86; *p* < 0.001). There were no differences between the samples in sex distribution or educational level. All preclinical markers were assessed at baseline and the main outcome of interest was incident dementia across the 6-year follow-up.

The ethical committee at Karolinska Institutet and the regional ethical review board Stockholm, Sweden, have approved all parts of the SNAC-K project. All participants gave written informed consent. In cases of severe cognitive impairment, primarily individuals who were living in a nursing home, a proxy was asked for consent.

### Dementia diagnosis

All-cause dementia was clinically diagnosed according to DSM-IV criteria [30] at each wave, following a 2–h long clinical interview. Cognitive functioning was assessed by the MMSE, the Clock test [31] and items regarding memory, executive functioning, problem solving, orientation, and interpretation of proverbs. The diagnosis of dementia followed a 3-step procedure that involved the examining physician, a physician who did not meet the participant and, in case of disagreement between the two, a supervising neurologist who made the final diagnosis. Death certificates and medical records were reviewed for those who died before receiving a dementia diagnosis in SNAC-K to identify additional dementia cases. Neuropsychological assessment, genotyping, and MRI were performed on a separate occasion and not included in the diagnostic process.

### Neuropsychological assessment

The neuropsychological test battery was administered following a standardized procedure [32]. The specific factors included measures from one of the five cognitive domains (2 tasks/domain), whereas the global factor included all 10 measures.

*Perceptual speed* was assessed with digit cancellation [33] and pattern comparison [34]. *Episodic memory* was assessed with tests of free recall and recognition [32] and *semantic memory* with a vocabulary [35] and a general knowledge [36] test. For verbal fluency [37], participants were asked to generate as many words as possible in 60 s. The test involved both *letter* (F, A) and *category* (animals, professions) *fluency*.

### Genotyping

DNA was extracted from peripheral blood samples using standard methods. Genotyping was performed using MALDI-TOF analysis on the Sequenom MassARRAY platform at the Mutation Analysis Facility, Karolinska Institutet [38] and *APOE* genotype results were in Hardy-Weinberg equilibrium (*p* > 0.05). *APOE* status was coded as 1 (any *ɛ*4) or 0 (no *ɛ*4 allele) and, as the genetic information only included *APOE*, this variable was entered in both the global and specific modelling.

### Brain imaging assessment

Brain scans were performed on a 1.5T MRI scanner (Philips Intera, The Netherlands). The protocol included an axial 3D T1-weighted fast field echo with repetition time (TR) = 15 ms, echo time (TE) = 7 ms, flip angle = 15°, field of view (FOV) = 240, 128 slices with 1.5 mm thickness and in-plane resolution of 0.94×0.94 mm, no gap, matrix 256×256, and an axial turbo fluid attenuation inversion recovery (FLAIR) sequence; TR = 6000 ms, TE = 100 ms, inversion time = 1900 ms, flip angle = 90°, echo train length = 21, FOV = 230, 22 slices with 5 mm thickness and in-plane resolution of 0.90×0.90 mm, gap = 1 mm, and matrix 256×256.

For the DTI images, a single-shot diffusion-weighted echoplanar imaging sequence with the following parameters was conducted: FOV = 230×138 mm^2^, 128×77 matrix, TE = 104 ms, TR =6838 ms, slice thickness 5 mm with 1 mm gap and *b*-value 600 s/mm^2^. A DTI scheme with six non-collinear diffusion-weighting gradient directions was used to determine the diffusion tensor set.

### Brain imaging processing (T1, FLAIR)

The processing of the structural brain volume images was conducted using SPM12b software (Statistical Parametric Mapping, https://www.fil.ion.ucl.ac.uk/spm/), implemented in Matlab R2012b (The MathWorks Inc.). We used the unified segmentation approach [39] to segment grey matter, white matter, and cerebrospinal fluid. Volumes were further assessed for total grey matter, total white matter, total brain tissue (grey + white matter), and total intracranial volume (TIV: grey + white matter +cerebrospinal fluid). The Freesurfer image analysis suite version 5.1 (Martinos Center for Biomedical Imaging, Harvard-Massachusetts Institute of Technology) was used to measure hippocampal volume through automatic volumetric segmentation as previously described [40]. WMH were manually delineated on the FLAIR images [41].

All volumes were corrected for TIV using the analysis of covariance approach [42]. Total brain tissue volume was used for the creation of global models, whereas grey- and white-matter volumes, hippocampal volume, and WMH volume were used in forming the specific models.

### Diffusion tensor imaging processing

The DTI images were pre-processed using an iterative optimization algorithm for the diffusion tensor calculation. In the next step, fractional anisotropy (FA) and mean diffusivity (MD) were derived on a voxel-by-voxel basis using the approach from [43]. Further processing of the FA data was conducted using the tract-based spatial statistics (TBSS) tool of the FMRIB Software Library Analysis Group (FMRIB, Oxford, UK) [44]. Fourteen masks, one for each tract of interest in both hemispheres, were created and used to extract the FA and MD values of each participant as previously described [28]. These tracts were the cingulum cingulate gyrus (CCG), the portion of cingulum that extends to the hippocampus (CHC), the corticospinal tract (CS), the forceps major (FMAJ), the forceps minor (FMIN), the inferior fronto-occipital fasciculus (IFOF), and the superior longitudinal fasciculus (SLF).

For both FA and MD, a global (including all tracts) and tract-specific factors were derived to be used in the global or specific models, respectively.

### Statistical analysis

Group differences in descriptive characteristics were assessed with t-tests and *χ*^2^-tests (dichotomous variables). Raw scores for the predictor variables were normally distributed with acceptable skewness and kurtosis. Baseline group differences in the predictor variables were examined with a univariate analysis of covariance (ANCOVA), using age, sex, and education as covariates.

**Fig. 1:**
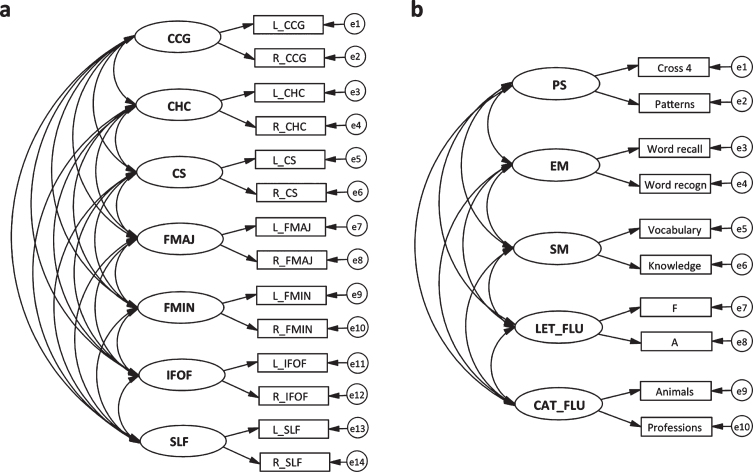
Graphical representations of structural equation models for 7 specific latent microstructural white matter integrity factors (a) and 5 specific latent cognitive factors (b). The same model applies to FA and MD. Latent factors are depicted with circles, endogenous variables with rectangles, regressions with one-headed arrows, and covariance with two-headed arrows. CCG, cingulum cingulate gyrus; CHC, cingulum hippocampus; CS, corticospinal tract; FMAJ, forceps major; FMIN, forceps minor; IFOF, inferior fronto-occipital fasciculus; SLF, superior longitudinal fasciculus; PS, perceptual speed; EM, episodic memory; SM, semantic memory; LET_FLU, letter fluency; CAT_FLU, category fluency. Adapted from Laukka et al. [15].

Latent factors were generated for the cognitive domains and white matter tracts using structural equation modelling (SEM). These procedures have been described and validated in previous publications [28, 32]. The SEM models for the white matter integrity (a) and cognition (b) modality are displayed in Fig. 1. All three models (DTI MD, DTI FA, and cognition) have a good fit according to the CFI (> 0.95) and RMSEA (< 0.08). The SEMs with global factors had a poorer model fit than the specific models [28, 32], but were still included to allow comparisons with other studies. For the DTI SEM models, the latent factors were formed by the left and right portion of each tract. This approach was based on previous observations of high correlations between left and right white-matter indicators in homologous tracts [28, 45].

All non-dichotomous variables were standardized, and some were reversed so that the odds ratio would always represent increased risk per SD-unit change in the predictor variable. This applied to the following variables: education, all cognitive variables, all brain volume (T1) and FA variables (see Table 2 for details). Pairwise associations between all specific and global variables were assessed with Pearson’s correlations. The dementia prediction models were created using multinomial logistic regressions in the IBM SPSS Statistics software version 24.0 (IBM Corporation) and receiver operating characteristics (ROC) analyses. In the regression analyses, three outcomes were possible: 1) no dementia (reference group), 2) incident dementia, and 3) death. The third outcome was included to account for the competing risk of death. However, as the outcome of interest was dementia, only the results for the reference and incident dementia groups are reported in this paper. Age, sex, and education were included as covariates in all models. The older participants (≥78 years at baseline) took part in dementia assessment both at three and six years. However, because we did not have information on dementia status for everyone at the three-year follow-up, time to diagnosis was not considered in the analyses.

ROC analyses were performed for the incident dementia versus no dementia outcome from the estimated probabilities of the multinomial logistic regressions. The resulting area under the curve (AUC) indicates how well people can be classified as demented versus non-demented; when choosing between competing models, the AUC was used as selection criterion. As an additional estimate of model fit, we report the Bayesian information criterion (BIC) [46]. To assess statistically significant differences in AUC between models, we calculated the DeLong’s test using the pROC package in R [47].

In the first step of the analysis, the impact of each individual marker in detecting future dementia was assessed. Subsequently, every possible variable combination for models containing two, three, and four predictors was assessed to ascertain the best model at each step. Modelling was terminated when the maximum number of predictors that could still add unique information to the model had been reached. Separate models were created for global and specific markers.

## RESULTS

Baseline characteristics of the no dementia and incident dementia groups are shown in Table 1. The no dementia group was significantly younger, had more years of education, and scored higher on the MMSE (*p* < 0.01). Raw scores and group differences for all predictor variables are shown in Supplementary Table 1. Correlations among all variables included in the statistical modelling are available in Supplementary Tables 2 and 3.

**Table 1:** Baseline characteristics according to dementia status at follow-up

*N*	No dementia	Incident dementia	*P*
	173	16
Age, y mean (SD)	69.76 (8.53)	78.05 (5.96)	< 0.001
Sex, *n* (%) female	113 (65.30)	12 (75.00)	0.613
Education, y mean (SD)	12.76 (3.65)	9.78 (3.08)	0.002
MMSE mean (SD)
Baseline	29.28 (0.87)	27.88 (1.45)	0.002
Follow-up	28.43 (1.44)	21.71 (2.50)	< 0.001
*APOE*
0 *ɛ*4, %	71.18	37.50	0.013
1 *ɛ*4, %	24.70	56.25
2 *ɛ*4, %	4.12	6.25
Hypertension (sbp≥140 or dbp≥90), %	58.58	78.57	0.17
Diabetes [54], %	6.36	12.50	0.30
Atrial fibrillation, %	10.40	6.25	0.99
Heart failure, %	3.47	12.50	0.14
Ischemic heart disease [55], %	12.72	18.75	0.45

NOTE. Group differences were assessed with independent t- or chi-square-tests. MMSE, Mini-Mental State Examination; sbp, systolic blood pressure; dbp, diastolic blood pressure.

### Individual markers

The results from the multinomial logistic regressions for individual markers are displayed in Table 2. For the cognitive modality, episodic memory, category fluency, and perceptual speed, as well as global cognition (AUC = 0.863–0.878) were most predictive of future dementia after accounting for the covariates. Genetic (*APOE*: AUC = 0.857) and brain volume variables (total brain tissue volume: AUC = 0.858, hippocampal volume: AUC = 0.857) were also strongly associated with incident dementia. For the DTI modality, the MD latent factors CHC, CS, FMAJ, IFOF, and the global MD factor (AUC-values = 0.837–0.862) were significant predictors of future dementia. Among the FA latent factors, only IFOF (AUC = 0.839) was significantly associated with dementia at six years. It should be noted, however, that if we were to apply strict corrections for multiple testing, many of the observed associations would not remain significant.

**Table 2:** Multinomial logistic regressions and ROC analyses for individual markers

	No dementia (*n*)	Incident dementia (*n*)	OR	95% CI for OR		*P*	ROC –AUC^a^
				Lower	Upper
Covariates
Age	173	16	2.918	1.570	5.422	0.001	0.765
Sex (female versus male)	173	16	1.593	0.492	5.153	0.437	0.548
Education	173	16	2.581	1.350	4.935	0.004	0.736
Combined	173	16					0.816
Cognitive
Global	173	16	3.675	1.680	8.039	0.001	0.878
Perceptual speed	171	15	3.784	1.574	9.097	0.003	0.864
Episodic memory	172	16	3.041	1.489	6.210	0.002	0.865
Semantic memory	173	16	1.865	1.078	3.226	0.026	0.852
Letter fluency	173	16	1.621	0.829	3.170	0.158	0.824
Category fluency	173	16	3.433	1.473	8.002	0.004	0.863
Genetic
*APOE* (any *ɛ*4 versus no *ɛ*4)	170	16	6.093	1.846	20.108	0.003	0.857
Brain volume (T1)
Total brain tissue volume	173	16	3.949	1.517	10.277	0.005	0.858
Grey matter volume	173	16	1.879	0.844	4.182	0.122	0.827
Hippocampal volume	170	15	2.692	1.259	5.754	0.011	0.857
White matter volume	173	16	1.940	1.015	3.707	0.045	0.826
Macrostructural white matter integrity (FLAIR)
WMH volume	167	16	1.941	0.975	3.866	0.059	0.841
Microstructural white matter integrity (DTI)
MD
Global	173	16	2.167	1.108	4.238	0.024	0.846
CCG	173	16	1.453	0.726	2.908	0.292	0.824
CHC	173	16	2.513	1.268	4.977	0.008	0.837
CS	173	16	2.104	1.147	3.859	0.016	0.862
FMAJ	173	16	2.373	1.264	4.456	0.007	0.853
FMIN	173	16	1.808	0.919	3.560	0.087	0.830
IFOF	173	16	2.193	1.159	4.149	0.016	0.851
SLF	173	16	1.663	0.911	3.036	0.098	0.837
FA
Global	173	16	1.618	0.866	3.024	0.131	0.837
CCG	173	16	1.823	0.943	3.524	0.074	0.844
CHC	173	16	1.353	0.681	2.689	0.387	0.818
CS	173	16	1.351	0.757	2.411	0.308	0.828
FMAJ	173	16	1.803	0.996	3.263	0.052	0.844
FMIN	173	16	1.541	0.826	2.876	0.174	0.832
IFOF	173	16	1.940	1.015	3.710	0.045	0.839
SLF	173	16	1.096	0.630	1.908	0.745	0.816

^a^no dementia versus incident dementia; Note: Age, sex, and education were included as covariates in all predictor models. OR, odds ratio; CI, confidence interval; ROC, receiver operating characteristics; AUC, area under the curve; WMH, white matter hyperintensities; DTI, diffusion tensor imaging; FA, fractional anisotropy; MD, mean diffusivity; CCG, cingulum cingulate gyrus; CHC, cingulum hippocampus; CS, corticospinal tract; FMAJ, forceps major; FMIN, forceps minor; IFOF, inferior fronto-occipital fasciculus; SLF, superior longitudinal fasciculus.

### Combined models: Global variables

Global cognition, in combination with the covariates, reached the highest AUC (0.878) in this study. Systematically exploring all possible combinations of global variables, we found that global cognition together with *APOE* rendered the best two-variable model for predicting future dementia (AUC = 0.900). The best three-variable model was obtained by adding total brain tissue volume to global cognition and *APOE* (AUC = 0.920, see Table 3). After this point, no marker added unique information. Adding global cognition to the covariate model did not lead to a significant increase in predictivity. However, the two-and three-variable models were significantly more predictive than the model including only the covariates (DeLong’s *p* < 0.040 and *p* < 0.006, respectively).

**Table 3:** Multinomial logistic regressions and ROC analyses for global models

		No dementia (*n*)	Incident dementia (*n*)	OR	95% CI for OR		*P*	BIC	ROC –AUC^a^	*p* DeLong’s^b^
					Lower	Upper
Model 0	Covariates	173	16					252.385	0.816
Model 1	Global cognition	173	16	3.675	1.680	8.039	0.001	250.904	0.878	0.102
Model 2	Global cognition	170	16	3.797	1.551	9.295	0.003	240.492	0.900	0.040
Any *ɛ*4 versus no *ɛ*4				5.470	1.541	19.415	0.009
Model 3	Global cognition	170	16	3.258	1.304	8.140	0.011	246.647	0.920	0.006
Any *ɛ*4 versus no *ɛ*4				4.849	1.348	17.450	0.016
Total brain tissue volume				3.024	1.043	8.768	0.042

^a^no dementia versus incident dementia; ^b^ compared to model 0; OR, odds ratio; CI, confidence interval; BIC, Bayesian information criterion; ROC, receiver operating characteristics; AUC, area under the curve.

### Combined models: Specific variables

The models of specific markers with the best likelihood to predict future dementia are displayed in Table 4. The best individual predictor was episodic memory and the best combination of two specific variables was obtained with episodic memory and *APOE* (AUC = 0.910). A model combining hippocampal volume, perceptual speed, and MD forceps major reached the highest AUC-value (0.911) among the specific models. That said, the difference in predictivity compared to the two-variable model was very minor. In addition, a three-variable model with hippocampal volume, category fluency, and *APOE* reached a similar AUC value (AUC = 0.910). There was, however, an effect of combining several markers from different modalities as the model with two (*p* = 0.007) and three (*p* = 0.004) variables accounted for significantly more variance than the covariate model.

**Table 4:** Multinomial logistic regressions and ROC analyses for specific models

		No dementia (*n*)	Incident dementia (*n*)	OR	95% CI for OR		*P*	BIC	ROC –AUC^a^	*p* DeLong’s^b^
					Lower	Upper
Model 0	Covariates	173	16					252.385	0.816
Model 1	Episodic memory	172	16	3.041	1.489	6.210	0.002	251.903	0.865	0.166
Model 2	Episodic memory	169	16	3.087	1.489	6.403	0.002	239.291	0.910	0.007
	Any *ɛ*4 versus no *ɛ*4			6.874	1.923	24.578	0.003
Model 3	Perceptual speed	168	14	2.667	1.042	6.829	0.041	244.869	0.911	0.004
	Hippocampal volume			2.452	1.030	5.838	0.043
	MD FMAJ			2.096	1.003	4.380	0.049

^a^no dementia versus incident dementia; ^b^compared to model 0; OR, odds ratio; CI, confidence interval; BIC, Bayesian information criterion; ROC, receiver operating characteristics; AUC, area under the curve; MD, mean diffusivity; FMAJ, forceps major.

Within the white matter integrity modality, MD in the corticospinal tract yielded the highest predictive value, higher than both MD global and MD forceps major. Nevertheless, MD forceps major performed best in combination with the other preclinical markers.

Controlling for hypertension in the final models had very little impact on the results. However, in the combined model for specific variables, perceptual speed was no longer significantly associated with incident dementia.

## DISCUSSION

This study shows that a combination of several markers improves the detection of individuals with a high probability to develop dementia. The results also suggest a benefit of combining information from several sources (i.e., cognitive, genetic, MRI), which is in line with previous studies [25, 48]. Several microstructural white matter integrity indicators were significantly associated with future dementia when tested individually. One of these, MD forceps major, was also included in the model with the highest predictive value. However, the DTI markers explained relatively little additional variance compared to the more established markers of preclinical dementia.

### Individual markers

The best single predictors belonged to the cognitive modality, which supports findings from previous studies [48, 49]. The highest overall AUC value (0.878) was obtained from the global cognition variable, including data from several cognitive domains. In general, the global measures showed high predictivity of future dementia suggesting that when screening for individuals with increased risk of dementia, having access to global measures may be a useful first step. Among the specific variables, episodic memory displayed the highest predictive value, closely followed by perceptual speed and category fluency. These measures have previously been found to be impaired in the preclinical phase of dementia [4, 48]. The modality with the second highest predictive value was DTI-derived white matter integrity, where MD of the corticospinal tract reached a higher AUC value than any other variable from the neuroimaging modalities. Also, MD of forceps major and inferior fronto-occipital fasciculus were significantly associated with future dementia. This is in line with other studies, showing that the corpus callosum and inferior fronto-occipital fasciculus show lower microstructural white matter integrity in persons at risk of AD [21, 22]. Interestingly, several tracts were more strongly associated with future dementia than WMH, suggesting that measures of microstructural white matter integrity may capture brain changes that are not detected with more commonly used macrostructural MRI sequences [13, 17, 19].

Previous studies [9, 26, 50] reported hippocampal volume as a strong predictor of future AD, likely due to the typical AD pattern of neuronal loss starting in the medial temporal lobe. Also in this study, hippocampal volume was associated with future dementia, as was being an *APOE* *ɛ*4 carrier. Note, however, that none of the individual markers were significantly more predictive than the covariate model, including age, sex, and education, and that more than one predictor was needed to reach a significant increase in AUC value. This is likely related to the small sample size, but also illustrates that having access to several preclinical markers allows for a better estimate of future dementia risk.

### Combined models: Global variables

The model combining global cognition, *APOE*, and total brain tissue volume reached the highest predictive value in this study (0.920). This finding supports recent research [25, 27, 51, 52], which used variables from these modalities to predict future dementia. Moreover, these findings are in line with research regarding the biological background and typical development of dementia, especially AD. It was not possible to include the global variable of the fourth modality—microstructural white matter integrity. This was so despite the fact that some of the white matter integrity variables added unique information in the specific modelling. A possible explanation for this pattern of results is that, although a global factor may explain a significant portion of common variance across all tracts, it does not capture all variance in the specific tracts [28]. Thus, some tracts may show stronger association to future dementia than the global MD variable.

### Combined models: Specific variables

The specific model with the highest predictive value (0.911) was obtained by combining predictors from three different modalities: cognitive (perceptual speed), brain volume (hippocampal volume), and microstructural white matter integrity (MD forceps major). This finding extends results of previous studies regarding cognition and hippocampal volume [26, 48] by showing that microstructural white matter integrity adds unique information over and above the other two predictors. This is so despite high correlations between white matter integrity, brain volume, and cognition.

Overall, the obtained pattern of results supports the findings from previous studies [25, 27, 48] that inter-modality prediction modelling leads to higher predictive values. Moreover, the results indicate that microstructural white matter integrity may be a good marker of future dementia. To verify the predictive power of specific white matter tracts, future studies with larger samples and more sophisticated DTI sequences should be conducted. Such studies might also include differentiation among specific dementia types, as it has been suggested that different white matter tracts are affected in AD and VaD [53]. Furthermore, DTI markers might perform better in detecting individuals at risk for VaD compared to AD.

It should be emphasized that several specific models reached almost the same predictive value. Thus, several combinations of variables may be equally useful for detecting individuals in the prodromal phase. For example, within the cognitive modality, episodic memory, perceptual speed and category fluency rendered similar AUC values and either of those, or a combined global score, is likely to produce good predictivity.

### Study strengths and limitations

A main advantage of this study is that the participants are part of a population-based sample, which increases the generalizability of the results. Also, information on the preclinical markers was not used for diagnosing dementia, which minimized the risk of circularity in the diagnosis.

The explanatory power of the study results is limited by a small sample size of 16 incident dementia cases and lack of separation between specific dementia types. Thus, the results need to be validated in future studies based on larger samples. The analytical sample is slightly positively biased (in terms of age and cognitive performance) compared to the full SNAC-K sample; however, this likely leads to an underestimation of the effects.

### Implications

Prediction models including a combination of preclinical markers can be helpful for improving early detection of dementia and selecting individuals that may be targeted for dementia prevention initiatives or clinical trials. However, depending on the aim, it is important to weigh the added effort in terms of time spent and financial cost against improved prediction accuracy.

## Supplementary Material

Supplementary Material
