# Supplementary material for: Cognitive, Genetic, Brain Volume, and Diffusion Tensor Imaging Markers as Early Indicators of Dementia
Source: J Alzheimers Dis. 2020 Oct 13;77(4):1443–53. doi: 10.3233/JAD-200445 (PMC7683082; doi:10.3233/JAD-200445)
Supplement: Supplementary Material [file jad-77-jad200445-s001.pdf]

Supplementary Material

Cognitive, Genetic, Brain Volume, and Diffusion Tensor Imaging Markers as Early Indicators of Dementia

Supplementary Table 1. Raw scores and baseline group differences for all predictor variables

	No dementia (*n*)	Incident dementia (*n*)	No dementia M (SD)	Incident dementia M (SD)	*p*	Partial eta squared
**Cognitive**						
Global	173	16	0.01 (0.02)	-0.02 (0.02)	0.001	0.057
Perceptual Speed						
Digit cancellation	171	15	19.12 (4.23)	15.07 (2.49)	0.016	0.032
Pattern comparison	171	15	15.62 (3.47)	11.40 (3.69)	0.013	0.033
Episodic memory						
Word recall	172	16	7.53 (2.29)	5.06 (2.18)	0.004	0.045
Word recognition	172	15	7.38 (4.37)	5.00 (2.54)	0.181	0.010
Semantic memory						
General knowledge	172	16	7.12 (1.55)	6.06 (1.77)	0.076	0.017
Vocabulary	172	16	24.45 (3.78)	21.50 (3.74)	0.170	0.010
Letter fluency	173	16	15.19 (4.52)	13.03 (3.26)	0.484	0.003
Category fluency	173	16	20.43 (4.78)	15.53 (3.88)	0.049	0.021
**Genetic**						
APOE (any ε4 versus no ε4), *n* (%)	170	16	49 (28.82)	10 (62.50)	0.002	0.054
**Brain volume (T1)**						
Total brain tissue volume, ml	173	16	1067.25 (69.81)	987.05 (67.64)	0.016	0.031
Total grey matter volume, ml	173	16	556.95 (52.18)	513.31 (44.67)	0.251	0.007
Hippocampal volume, ml	170	15	3.80 (0.38)	3.34 (0.36)	0.003	0.047
Total white matter volume, ml	173	16	510.30 (42.65)	473.79 (47.86)	0.081	0.016
**Macrostructural white matter integrity (FLAIR)**						
WMH volume, ml	167	16	0.75 (1.40)	2.04 (1.49)	0.076	0.018

**Microstructural white matter integrity (DTI)**						
MD						
Global	173	16	81.61 (3.80)	86.14 (4.52)	0.006	0.041
CCG	173	16	81.64 (4.11)	84.20 (3.27)	0.713	0.001
CHC	173	16	97.85 (8.13)	107.26 (10.81)	0.012	0.034
CS	173	16	74.36 (2.29)	77.37 (3.37)	0.001	0.055
FMAJ	173	16	76.90 (4.23)	82.11 (7.08)	0.003	0.047
FMIN	173	16	80.58 (5.37)	85.03 (5.45)	0.142	0.012
IFOF	173	16	83.25 (3.80)	87.46 (4.68)	0.009	0.037
SLF	173	16	76.66 (3.72)	79.55 (4.23)	0.161	0.011
FA						
Global	173	16	48.05 (2.16)	46.23 (1.82)	0.156	0.011
CCG	173	16	40.79 (2.77)	38.15 (2.48)	0.048	0.021
CHC	173	16	40.10 (2.60)	38.96 (2.72)	0.936	0.000
CS	173	16	56.43 (2.30)	54.98 (1.78)	0.258	0.007
FMAJ	173	16	58.02 (2.74)	55.71 (2.70)	0.075	0.017
FMIN	173	16	52.04 (3.21)	49.57 (3.28)	0.285	0.006
IFOF	173	16	47.04 (2.29)	44.88 (2.05)	0.043	0.022

WMH, white matter hyperintensities; DTI, diffusion tensor imaging; FA, fractional anisotropy; CCG, cingulum cingulate gyrus; CHC, cingulum hippocampus; CS, corticospinal tract; FMAJ, forceps major; FMIN, forceps minor; IFOF, inferior fronto-occipital fasciculus; SLF, superior longitudinal fasciculus; MD, mean diffusivity.

Supplementary Table 2. Correlations among global variables

		Any ε4	Total brain tissue volume	Global MD
Global cognition	Pearson correlation (*n*)	-0.021 (206)	0.483*** (212)	-0.383*** (212)
Any ε4	Pearson correlation (*n*)	-	0.045 (206)	0.025 (206)
Total brain tissue volume	Pearson correlation (*n*)		-	-0.659*** (212)

***$p < 0.001$; MD, mean diffusivity.

Supplementary Table 3. Correlations among specific variables

		SM	Cat_Flu	PS	Any ε4	Hippoc. volume	WM volume	MD CHC	MD CS	MD FMAJ	MD IFOF
EM	Person correlation	0.432***	0.577***	0.506***	0.050	0.338***	0.234**	-0.238***	-0.246***	-0.263***	-0.244***
	(*n*)	(211)	(211)	(208)	(205)	(205)	(211)	(211)	(211)	(211)	(211)
SM	Person correlation	-	0.665***	0.577***	-0.068	0.250***	0.216**	-0.262***	-0.246***	-0.249***	-0.241***
	(*n*)		(212)	(208)	(206)	(206)	(212)	(212)	(212)	(212)	(212)
Cat_Flu	Person correlation		-	0.783***	-0.006	0.373***	0.353***	-0.401***	-0.372***	-0.392***	-0.382***
	(*n*)			(208)	(206)	(206)	(212)	(212)	(212)	(212)	(212)
PS	Person correlation			-	-0.084	0.444***	0.458***	-0.509***	-0.484***	-0.503***	-0.489***
	(*n*)				(202)	(202)	(208)	(208)	(208)	(208)	(208)
Any ε4	Person correlation				-	-0.026	-0.058	0.002	0.067	0.012	0.017
	(*n*)					(200)	(206)	(206)	(206)	(206)	(206)
Hippoc. vol.	Person correlation					-	0.431***	-0.541***	-0.512***	-0.528***	-0.523***
	(*n*)						(206)	(206)	(206)	(206)	(206)
WM vol.	Person correlation						-	-0.584***	-0.421***	-0.533***	-0.467***
	(*n*)							(212)	(212)	(212)	(212)
MD CHC	Person correlation							-	0.671***	0.892***	0.802***
	(*n*)								(212)	(212)	(212)
MD CS	Person correlation								-	0.892***	0.891***
	(*n*)									(212)	(212)
MD FMAJ	Person correlation									-	0.966***
	(*n*)										(212)

**$p < 0.01$, ***$p < 0.001$.

EM, episodic memory; SM, semantic memory; Cat_Flu, category fluency; PS, perceptual speed; Hippoc. vol., hippocampal volume; WM vol., white matter volume; MD, mean diffusivity; CHC, cingulum hippocampus; CS, corticospinal tract; FMAJ, forceps major; IFOF, inferior fronto-occipital fasciculus.
